# Case Report: Histological study of gastric leiomyosarcoma in a young horse

**DOI:** 10.3389/fvets.2026.1979698

**Published:** 2026-10-21

**Authors:** Hernán Guerrero, Belén Varela, Agustín Morales, Camila Larrañaga, Victoria Yozzi, Kanji Yamasaki, José Manuel Verdes

**Affiliations:** 1 Academic Unit of Equine Medicine and Surgery, Department of Veterinary Clinics and Hospital, Faculty of Veterinary, Universidad de la República, Montevideo, Uruguay; 2 Academic Unit of Pathology, Department of Pathobiology, Faculty of Veterinary, Universidad de la República, Montevideo, Uruguay

**Keywords:** horse, leiomyosarcoma, pathology, stomach, tumor

## Abstract

During the necropsy of a 4-year-old male horse, a protruding mass was detected in the nonglandular region of the stomach. It was roughly oval in shape with a brown surface and a grayish-white cut surface. The tumor was attached to part of the stomach wall. Histologically, the tumor cells were derived from the smooth muscle layer. Tumor cells showed various morphologies and nuclear atypia. Mitotic figures were rare. Some of the tumor cells resembled smooth muscle cells. Tumor cells were seen in blood vessels within the tumor. Immunohistochemically, the tumor cells were positive for alpha smooth muscle actin antibody and were negative for CD117 (c-kit) antibody. Based on these findings, the tumor was diagnosed as leiomyosarcoma derived from gastric smooth muscle.

## 1 Introduction

The stomach is the most commonly affected organ by gastrointestinal neoplasms in horses. However, gastric neoplasms are relatively rare in equines, with an incidence of 0.9% (1, 2). There are few reports on tumors in the stomach of herbivores (3–6). Gastric neoplasms in horses typically present at a mean age of 18 years, showing no significant correlation with breed or sex (1, 2). While squamous cell carcinoma is the most frequently diagnosed form, muscle-derived tumors are exceptionally rare, despite a documented case of gastric leiomyoma in a 2-year-old horse (7). Other reported gastric tumors include mesothelioma, adenocarcinoma, lymphoma, and leiomyoma -the latter observed in two out of 24 horses in one series (6). Conversely, leiomyosarcoma is extremely scarce, with only one documented case in a 12-year-old horse diagnosed through H&E morphological evaluation (3). Furthermore, differential diagnosis between leiomyosarcoma and gastrointestinal stromal tumor (GIST) is considered necessary in various species, including humans, and immunohistochemical testing has been shown to be crucial alongside morphological assessment by Hematoxylin-Eosin (H&E) staining (2, 4, 8–12). For example, an immunohistochemical reexamination of 42 canine gastrointestinal tumors previously diagnosed as leiomyosarcoma revealed that only 10 were confirmed as leiomyosarcoma, with the remaining cases identified as GIST or undifferentiated sarcoma (12). Therefore, both morphological examinations using H&E staining and immunohistochemical analysis are essential for the accurate diagnosis of gastrointestinal smooth muscle tumors.

The diagnosis of gastrointestinal stromal tumors (GIST) heavily relies on immunohistochemical staining for KIT receptor tyrosine kinase (CD117) (KIT/CD117), which detects the majority of these tumors, except for approximately 3%−5% that harbor PDGFRA mutations (13). Conversely, immunohistochemical expression of alpha-smooth muscle actin (α-SMA) is indicative of gastric leiomyosarcoma (14). The survival time following the appearance of clinical signs in horses with gastric tumors is typically short, approximately 4 weeks, often culminating in euthanasia or death of the animal (5, 6).

The aim of this study was to characterize the histopathological (H&E staining) and immunohistochemical features of a leiomyosarcoma arising in the nonglandular region of the stomach of a 4-year-old horse.

## 2 Case description

A 4-year-old stallone male Thoroughbred racehorse used for flat racing presented in August 2023 with a history of intermittent low-grade fever, mild inappetence following competition, and subtle weight loss. The horse had no prior history of gastrointestinal disease and maintained a generally bright demeanor with preserved appetite and fecal consistency throughout the early stages of the clinical course.

Over a period of approximately 3 months (August to November 2023), the horse underwent extensive diagnostic evaluation due to recurrent febrile episodes (ranging from 38.5 °C to 40 °C), gradual weight loss (~25 kg), and persistent hypoalbuminemia. Physical examinations revealed no remarkable abnormalities aside from episodes of pyrexia and a well-organized jugular phlebitis that developed secondary to repeated venipuncture. Auscultation, thoracic ultrasonography, and respiratory parameters remained within normal limits, and no cough, nasal discharge, or respiratory distress were observed at any stage.

Serial blood work complete blood count (CBC and serum biochemistry) revealed progressive hypoproteinemia and hypoalbuminemia without leukocytosis. Parasitological fecal analysis revealed a low load of strongyle-type eggs (20 EPG). Thoracic and abdominal ultrasonography showed transient splenomegaly and mild pleural irregularities; no abdominal masses or effusions were detected. Bronchoalveolar lavage fluid (BALF) cytology showed 59% macrophages, 39% lymphocytes, and 2% eosinophils, with notable presence of activated macrophages and multinucleated giant cells—consistent with chronic inflammatory changes. No intracellular or extracellular bacteria were detected. Urine cultures, head radiographs, and repeated viral/hemoparasitic screenings were negative.

Gastroscopy was performed approximately 1 week prior to death. Despite a 24-h fasting period, incomplete gastric emptying was noted. The gastric mucosa, however, appeared macroscopically normal, and no masses, ulcerations, or active bleeding were observed. Given the persistent clinical picture, the horse was placed on antimicrobial therapy (initially ceftiofur, followed by enrofloxacin and metronidazole), supportive care, and intravenous flunixin meglumine as needed for the management of pyrexia.

On November 16, the horse acutely developed higher fever (up to 40 °C), mild depression, and decreased feed intake. Thoracic radiographs revealed a bronchial-interstitial pattern, bronchiectasis, and a large, well-defined structure consistent with a pulmonary abscess. The horse was euthanized and referred for necropsy to the Pathology Unit, Department of Pathobiology, Faculty of Veterinary, Universidad de la República (< city>Montevideo < /city>, Uruguay).

At necropsy, a large intramural mass was discovered protruding from the gastric wall of the nonglandular gastric region and was found within the ingested feed (Figure 1A). The mass was generally oval in shape and measured 20 × 11 × 11cm. The surface was brown in color and the hardness was soft. The cut surface was grayish white with scattered black areas. Necrosis was observed in some parts. The base of the tumor was firmly adhered to the stomach wall, and the stomach wall was thickened (Figure 1B). A mass was fixed in 10% buffered neutral formalin solution and routinely processed for histological examination. Tissue sections were stained with H&E. Additional 3 μm serial sections were prepared for immunohistochemistry. The following primary antibodies were used: alpha smooth muscle actin antibody (SMA, 1:150; Biocare Medical, USA) and CD117 (c-kit) antibody (1:100, Biocare Medical, Pacheco, California, USA).

**Figure 1:**
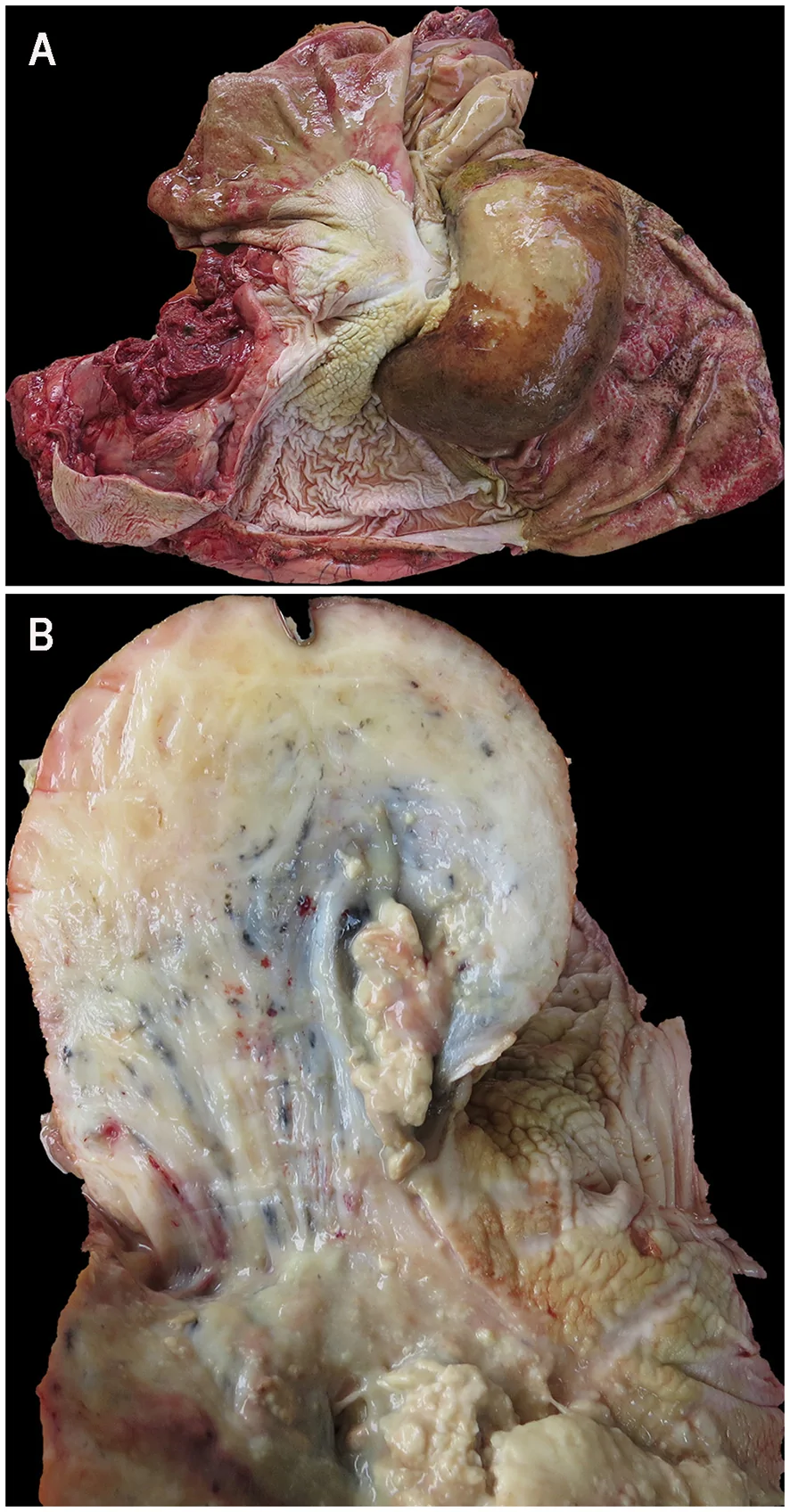
**(A)** Gastric leiomyosarcoma in a horse. A large tumor protruding into the gastric lumen. **(B)** Sagittal section of the gastric leiomyosarcoma in a horse.

Histologically, the tumor originated in close association with the normal smooth muscle layer (Figure 2A). In these regions, the typical architecture of smooth muscle was disrupted by neoplastic cell proliferation, connective tissue expansion, and interstitial edema. Infiltration of tumor cells, accompanied by inflammatory infiltrates and edema, was also observed in the serosal layer underlying the muscularis, resulting in serosal thickening (Figure 2B). A portion of the tumor surface that had protruded into the gastric lumen was covered by squamous epithelium; however, the majority of the surface exhibited extensive necrosis, accompanied by lymphocytic, plasmacytic, and histiocytic inflammatory responses. Remnants of ingesta and clusters of bacteria were present in some necrotic areas (Figure 2C).

**Figure 2:**
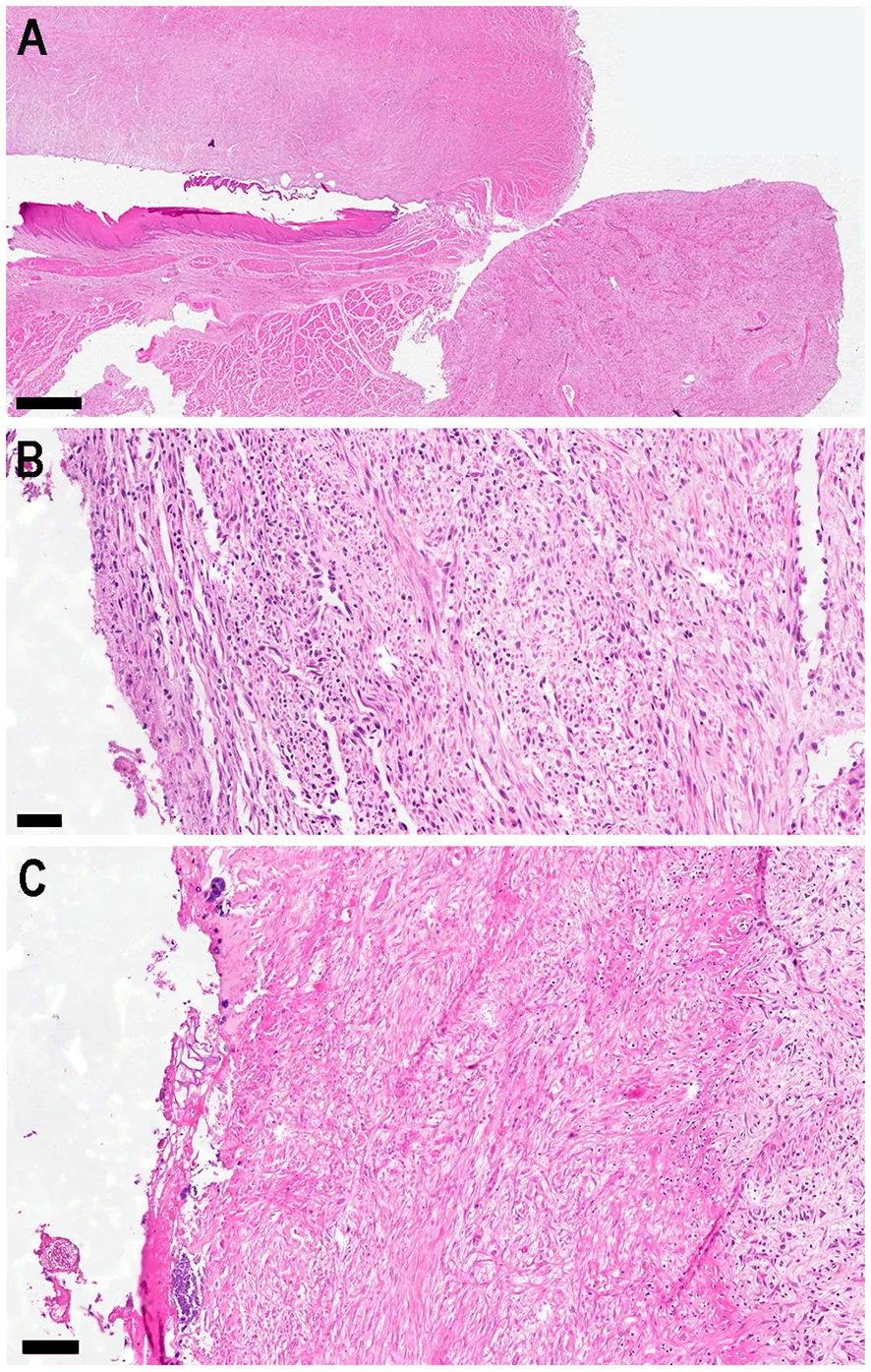
**(A)** Large tumor protruding into the stomach, observed in the histological section stained with hematoxylin and eosin (H&E), × 0.93. Scale bar = 1,600 μm. **(B)** Infiltration of tumor cells accompanied by inflammatory cells and edema in the serosal layer underlying the muscularis, resulting in thickening of the serosa (H&E), × 20. Scale bar = 50 μm. **(C)** Presence of ingested material remnants and bacterial clusters within necrotic areas (H&E), × 10. Scale bar = 100 μm.

Tumor cells exhibited considerable pleomorphism, including round, pleomorphic, and spindle-shaped forms, with cytoplasmic volume ranging from abundant to scant. Nuclei were round, oval, or irregularly shaped, and mitotic figures were infrequent (Figure 3A). Neoplastic cells were arranged predominantly in interwoven or interlacing fascicles (Figure 3B). In certain areas, spindle-shaped cells with abundant eosinophilic cytoplasm formed clusters reminiscent of smooth muscle differentiation. Neoplastic cells were also identified within newly formed intratumoral blood vessels (Figure 3C).

**Figure 3:**
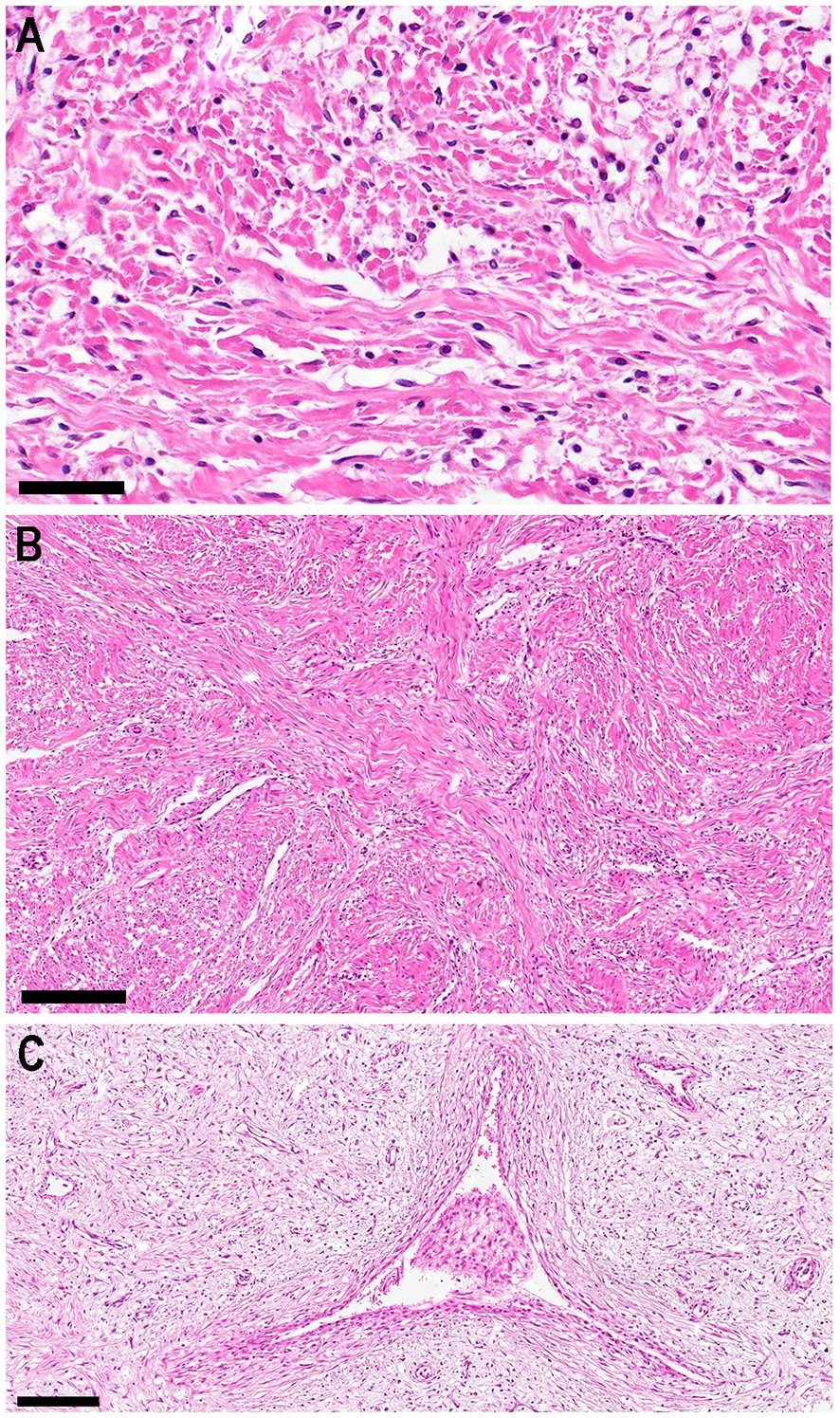
**(A)** Neoplastic cells predominantly arranged in interlaced or crossed fascicles, (H&E). × 10. Scale bar = 50 μm. **(B)** Tumor cells exhibiting morphological variability and atypia, (H&E). × 40. Scale bar = 200 μm. **(C)** Tumor cells within neovascular structures (H&E), × 10. Scale bar = 200 μm.

Immunohistochemically in serial histologic sections of the same specimen, the tumor cells exhibited strong cytoplasmic staining for smooth muscle actin (Figure 4A). Blood vessels showing positive labeling served as an internal positive control for the technique (Figure 4A). In contrast, the neoplastic cells were negative for CD117 (c-Kit; Figure 4B), to better understanding the tumor region of the serial immunohistochemical sections were compared with their serial section H&E-staining (Figure 4C). These findings are consistent with a final diagnosis of gastric leiomyosarcoma.

**Figure 4:**
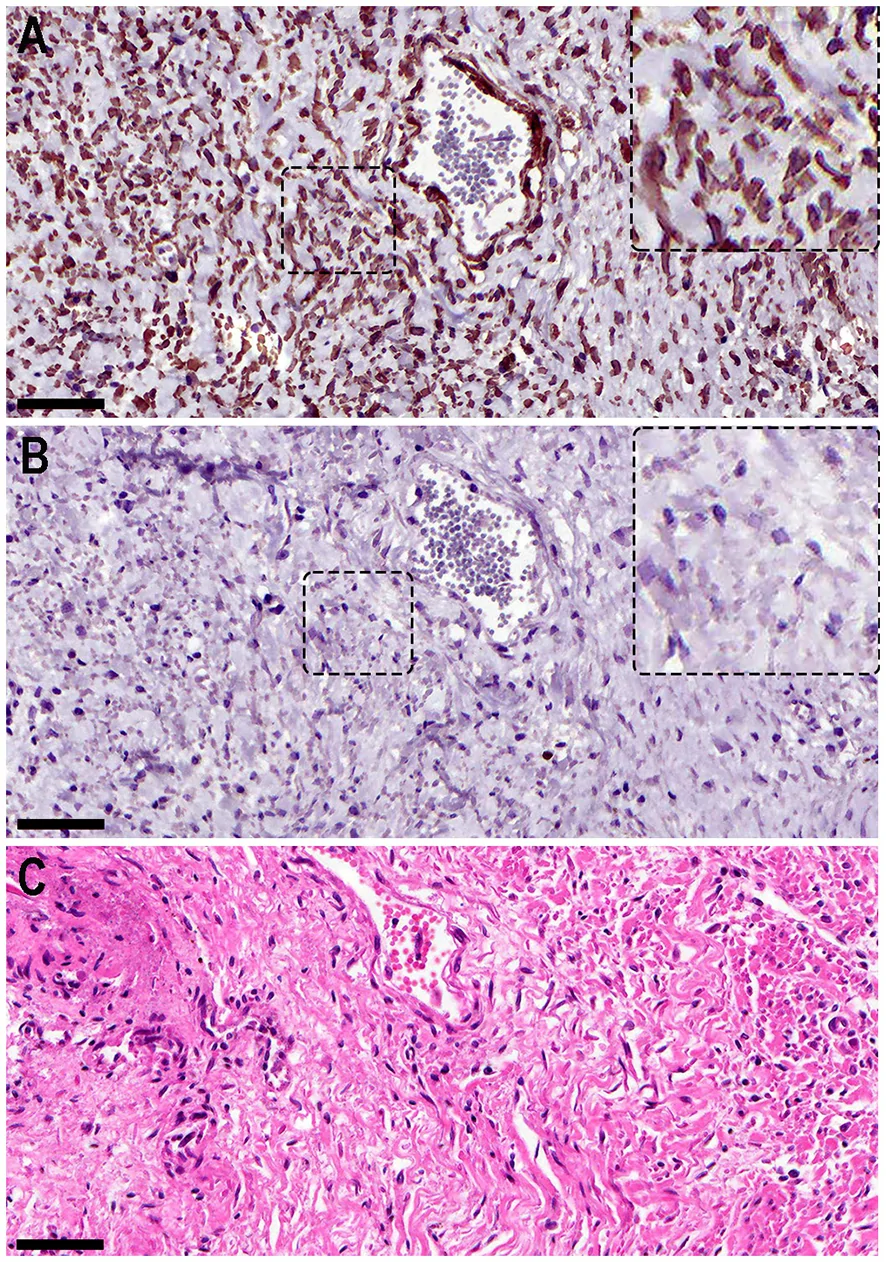
**(A)** Tumor cells positive for smooth muscle actin (SMA). A high magnification of dashed field is shown in the inset, × 20. Scale bar = 50 μm. **(B)** The same sample showing tumor cells negative for CD117 (c-kit). A high magnification of dashed field is shown in the inset, × 20. Scale bar = 50 μm. **(C)** H&E-stained section of the same sample, × 20. Scale bar = 50 μm.

No metastatic lesions were identified in the liver, lungs, or other examined organs. The diagnosis of primary gastric leiomyosarcoma was confirmed through histopathological and immunohistochemical evaluation. The tumor is presumed to have been responsible for the persistent fever, protein-losing state, and impaired gastric emptying observed prior to death.

## 3 Discussion

Smooth muscle-derived tumors in horses are uncommon. Reported cases include leiomyomas and leiomyosarcomas of the small and/or large intestines (5, 14, 15), as well as leiomyosarcomas of the urogenital and genital tracts (13, 15). Gastric tumors in horses are even rarer (2–6), and to the authors' knowledge, only two cases of gastric leiomyoma and a single case of gastric leiomyosarcoma have been described in horses (3, 6). Notably, the previously reported equine gastric leiomyosarcoma was diagnosed based solely on morphological findings, without immunohistochemical confirmation. In the present case, histological evaluation revealed tumor cells exhibiting variable morphology. However, spindle-shaped cells with abundant eosinophilic cytoplasm were present in several areas, and neoplastic proliferation was noted in direct association with the smooth muscle layer. These findings support a smooth muscle origin. In addition, nuclear atypia and vascular invasion confirmed the malignant nature of the neoplasm, leading to a diagnosis of leiomyosarcoma.

Gastrointestinal stromal tumors (GISTs) have also been reported in horses (16–18). Differentiating GISTs from smooth muscle tumors based on morphology alone can be challenging, particularly in canine gastric tumors (12). In the current case, immunohistochemical analysis demonstrated strong positivity for smooth muscle actin (SMA) and negativity for CD117 (c-Kit). Previous studies have proposed that gastrointestinal tumors with a SMA-negative/CD117-positive profile should be classified as GISTs, whereas those with a SMA-positive/CD117-negative profile are more consistent with leiomyomas or leiomyosarcomas (4). Similar immunoprofiles—SMA positivity and CD117/S100 negativity—have been reported in feline gastric leiomyosarcoma (8) and canine gastric leiomyosarcoma (11). Additionally, bovine luminal leiomyosarcoma has shown SMA immunopositivity (2). Together, these findings strongly support the diagnosis of gastric leiomyosarcoma in this case, based on both histopathological and immunohistochemical criteria. Gastrointestinal tumors, including those of the stomach, are exceedingly rare in young horses. The only other equine case of gastric leiomyosarcoma occurred in a 12-year-old horse (3, 6). Similarly, luminal leiomyosarcoma in cattle has been described in adult animals (2), and pyloric leiomyosarcomas in dogs and cats typically occur at 7 and 10 years of age, respectively (8, 11). The occurrence of a gastric leiomyosarcoma in a 4-year-old horse, as reported here, is therefore particularly noteworthy. Furthermore, in horses, smooth muscle tumors of the stomach tend to arise in the nonglandular region of the stomach (3, 6), consistent with the location of the tumor in this case. Although tumor cells were identified within intratumoral blood vessels, no evidence of metastatic spread was found in the lungs, liver, or other organs.

## 4 Conclusion

Histological and immunohistochemical examinations were performed on a nonglandular gastric region mass discovered at necropsy in a 4-year-old male horse. The combined morphological and immunophenotypic findings confirmed the diagnosis of gastric leiomyosarcoma originating from the smooth muscle layer of the non-glandular region of the stomach.

## Data Availability

The datasets presented in this article are not readily available because not applicable. No separate dataset was generated for this study. Requests to access the datasets should be directed to not applicable. No separate dataset was generated for this study.
